# Serum Interleukin-10 as a Potential Biomarker for Deep Remission and Histologic Activity in Ulcerative Colitis

**DOI:** 10.3390/biomedicines14040908

**Published:** 2026-04-16

**Authors:** Nikolaos Martinos, Christos Kroupis, Andreas C. Lazaris, Georgia-Eleni Thomopoulou

**Affiliations:** 1 Gastroenterology Department, Naval Hospital of Athens, 70 Dinokratous St., 115 21 Athens, Greece; 2 Department of Clinical Biochemistry, Attikon University General Hospital, National and Kapodistrian University of Athens, 124 62 Athens, Greece; ckroupis@med.uoa.gr; 3 First Department of Pathology, School of Medicine, National and Kapodistrian University of Athens, 115 27 Athens, Greece; alazaris@med.uoa.gr; 4 Cytopathology Department, Attikon University General Hospital, School of Medicine, National and Kapodistrian University of Athens, 124 62 Athens, Greece; gthomop@med.uoa.gr

**Keywords:** ulcerative colitis, interleukin-10, deep remission, endoscopic remission, histologic healing

## Abstract

**Background/Objectives:** Deep remission, defined as the coexistence of endoscopic remission and histologic healing, has emerged as an advanced therapeutic target in ulcerative colitis (UC). However, reliable non-invasive biomarkers capable of accurately reflecting histologic inflammatory status remain limited. We aimed to evaluate the association between serum interleukin-10 (IL-10) concentrations and deep remission in UC and to assess its discriminatory performance. **Methods:** In this prospective single-center observational study, consecutive adult patients with ulcerative colitis undergoing clinically indicated colonoscopy were enrolled. Serum IL-10 concentrations were measured prior to endoscopy using an enzyme-linked immunosorbent assay. Histologic healing was defined as a Geboes score < 2.0, and deep remission as the coexistence of endoscopic remission and histologic healing. Associations were evaluated using Firth penalized logistic regression. Discrimination was assessed using receiver operating characteristic analysis with bootstrap internal validation (500 resamples). **Results:** Among 44 patients, 18 (40.9%) achieved deep remission. Serum IL-10 levels were significantly higher in patients with histologic healing compared with those with active histologic inflammation (*p* < 0.001). Log-transformed IL-10 was independently associated with deep remission in both univariable and multivariable models. The primary multivariable model demonstrated apparent good discrimination (apparent AUC 0.88; optimism-corrected AUC 0.85). Among patients in endoscopic remission, non-detectable IL-10 identified persistent histologic activity with high sensitivity (92.9%) and negative predictive value (94.4%), although these findings are exploratory. **Conclusions:** Serum IL-10 concentrations were independently associated with deep remission and showed potentially promising internally validated discriminatory performance within this cohort. These findings are hypothesis-generating and require external validation in larger cohorts before clinical application.

## 1. Introduction

Inflammatory bowel disease (IBD), encompassing ulcerative colitis and Crohn’s disease, is characterized by chronic, immune-mediated intestinal inflammation with a relapsing and remitting course [1,2,3]. Over the past decade, therapeutic strategies have shifted from symptom-based management toward objective treatment targets within a treat-to-target framework. Endoscopic remission is now widely recognized as a major therapeutic goal and is associated with improved long-term outcomes.

However, increasing evidence indicates that endoscopic remission does not invariably reflect complete mucosal resolution [4,5]. Histologic activity may persist despite apparent mucosal healing and has been associated with increased risk of relapse, corticosteroid use, hospitalization, and disease progression [6,7]. Consequently, the concept of deep remission—defined as the concurrent presence of endoscopic remission and histologic healing—has emerged as a more stringent and potentially prognostically meaningful target [8,9,10].

Despite its clinical relevance, deep remission remains challenging to assess in routine practice. Histologic evaluation requires invasive procedures, and commonly used systemic inflammatory markers such as C-reactive protein (CRP) lack sufficient sensitivity to detect residual microscopic inflammation [11,12,13]. The identification of non-invasive biomarkers capable of reflecting mucosal immune resolution therefore represents an important unmet need.

Interleukin-10 (IL-10) is a central immunoregulatory cytokine that plays a pivotal role in maintaining intestinal immune homeostasis. IL-10 suppresses pro-inflammatory cytokine production, regulates macrophage activation, and limits T-cell–mediated immune responses [14,15]. Impaired IL-10 signaling has been implicated in severe intestinal inflammation, and genetic defects in IL-10 pathways are strongly associated with aggressive early-onset colitis. These observations underscore the critical role of IL-10 in intestinal immune regulation [16].

While previous studies have largely focused on pro-inflammatory mediators, the relationship between circulating IL-10 levels and the achievement of deep remission in established IBD has not been fully characterized. Moreover, it remains unclear whether IL-10 may help identify histologic activity among patients who have already achieved endoscopic remission, a clinically relevant scenario in contemporary disease monitoring.

Therefore, the aim of this study was to investigate the association between serum IL-10 concentrations and deep remission in a well-characterized ulcerative colitis cohort. We further evaluated the discriminatory performance of IL-10, its potential incremental value beyond conventional inflammatory markers, and its exploratory utility in detecting histologic activity within endoscopic remission [17,18].

## 2. Materials and Methods

### 2.1. Study Design and Participants

This was a prospective, single-center, observational cross-sectional study conducted at the Hepato-Gastroenterology Unit of the Naval Hospital of Athens, Greece. Consecutive adult patients (≥18 years) with an established diagnosis of ulcerative colitis (UC) who were scheduled to undergo clinically indicated colonoscopy were invited to participate. The diagnosis of UC was based on established clinical, endoscopic, radiologic, and histopathologic criteria.

Exclusion criteria included pregnancy, active infection, known malignancy, severe organ failure, antibiotic use within four weeks prior to enrollment, and recent initiation or modification of biologic therapy within four weeks, in order to avoid acute treatment-related effects on systemic cytokine signaling. Consecutive enrollment was applied to minimize selection bias.

The analyzed cohort included patients with ulcerative colitis only; no patients with IBD-unclassified/indeterminate colitis were present in the final analytic dataset.

The study was conducted and reported in accordance with the Strengthening the Reporting of Observational Studies in Epidemiology (STROBE) guidelines. The STROBE checklist is provided in Supplementary Table S1.

No missing data were observed for key study variables, including IL-10 concentration, histologic assessment, and CRP.

### 2.2. Ethical Approval

The study was conducted in accordance with the Declaration of Helsinki (1975, revised in 2013). The protocol was approved by the Institutional Review Board of the Naval Hospital of Athens (protocol code 3366, approved 11 April 2025) and by the Institutional Review Board of Attikon University Hospital (protocol code 219, approved 8 April 2025). Written informed consent was obtained from all participants prior to enrollment.

### 2.3. Blood Sampling and Processing

Peripheral venous blood samples were collected immediately prior to colonoscopy and before any endoscopic manipulation. Serum was separated by centrifugation within two hours of collection and stored at −80 °C until analysis. All samples were analyzed in batch during the same study period in order to minimize inter-assay variability. The median storage duration was approximately three months, and each sample underwent a single freeze–thaw cycle prior to cytokine quantification. Pre-analytical handling procedures were identical across all participants.

### 2.4. IL-10 Quantification

Serum interleukin-10 (IL-10) concentrations were measured using a commercially available sandwich enzyme-linked immunosorbent assay (Human IL-10 Quantikine ELISA Kit, D1000B; R&D Systems, Minneapolis, MN, USA) according to the manufacturer’s instructions. All samples and standards were measured in duplicate, and mean values were used for statistical analysis. Optical density was measured at 450 nm with wavelength correction using a Bio-Tek ELx800 microplate reader (BioTek Instruments, Inc., Winooski, VT, USA). Cytokine concentrations were calculated using four-parameter logistic curve fitting.

The lower limit of detection (LOD) was 3.9 pg/mL, and the dynamic quantifiable range was 7.8–500 pg/mL. Observed intra-assay coefficients of variation ranged from 5.3% to 7.2%, and inter-assay coefficients ranged from 8.2% to 10.1%, consistent with manufacturer specifications. Laboratory personnel performing IL-10 quantification were fully blinded to clinical data, endoscopic findings, histologic assessment, and remission status throughout sample processing and analysis.

### 2.5. Handling of Detection Limits

Values below the lower limit of detection (3.9 pg/mL) were imputed as half the detection limit (1.95 pg/mL), in accordance with established approaches for left-censored biomarker data in small observational cohorts. IL-10 concentrations were primarily analyzed as natural log-transformed (ln-transformed) continuous variables to reduce right-skewness and stabilize variance. Accordingly, odds ratios correspond to a one-unit increase in ln-transformed IL-10, representing a multiplicative increase in IL-10 concentration on the original scale.

To assess robustness, exploratory analyses additionally evaluated IL-10 detectability status (≥3.9 pg/mL versus <3.9 pg/mL). These analyses were considered supportive and did not replace the primary continuous modeling strategy.

### 2.6. Endoscopic and Histologic Assessment

Colonoscopy was performed according to standard clinical practice. Endoscopic activity in ulcerative colitis (UC) was assessed using the Mayo endoscopic subscore, and endoscopic remission was defined a priori as a Mayo score of 0 or 1.

Mucosal biopsies were obtained during colonoscopy and evaluated according to the Geboes scoring system by two experienced gastrointestinal pathologists who were blinded to cytokine data. In cases of discrepancy, consensus review was performed. Histologic healing was defined as a Geboes score < 2.0, consistent with prior studies in ulcerative colitis.

### 2.7. Outcome Definitions

The primary endpoint was deep remission, defined as the simultaneous presence of endoscopic remission and histologic healing (Geboes score < 2.0). All analyses for the primary endpoint were conducted in the ulcerative colitis cohort. The key secondary endpoint was histologic activity among patients in endoscopic remission.

### 2.8. Treatment Classification

At the time of colonoscopy, patients were categorized according to ongoing maintenance therapy, including no treatment, 5-aminosalicylic acid (5-ASA), advanced biologic therapy, or combination therapy consisting of biologic agents plus azathioprine. Treatment exposure was analyzed descriptively according to remission status. Given the potential for confounding, biologic therapy exposure was incorporated into the primary multivariable models. To preserve model stability, treatment was modeled as a binary variable (biologic vs. non-biologic therapy). To evaluate the impact of treatment adjustment, comparative analyses of models with and without treatment exposure were performed, with effect estimates for IL-10 remaining quantitatively stable (i.e., no meaningful change in odds ratio magnitude or direction).

All patients receiving biologic or combination therapy were on stable maintenance treatment, with no recent initiation or dose modification within four weeks prior to enrollment.

### 2.9. Laboratory Measurements

Serum C-reactive protein (CRP) concentrations were measured using standardized automated assays in the hospital’s accredited clinical laboratory.

### 2.10. Statistical Analysis

Continuous variables are presented as medians with interquartile ranges and were compared using the Mann–Whitney U test. Categorical variables are presented as counts and percentages and were compared using Fisher’s exact test or the χ^2^ test, as appropriate.

Associations between IL-10 and deep remission were evaluated using Firth penalized logistic regression in order to mitigate small-sample bias and address the anticipated risk of quasi-separation given the expected strength of the biomarker–outcome relationship. Firth penalization was prespecified due to the modest sample size. Three prespecified models were evaluated within the ulcerative colitis cohort: a univariable IL-10 model, a multivariable model including IL-10, CRP, biologic therapy, and disease extent (Montreal classification: E1/E2/E3), and an extended multivariable model including IL-10, CRP, biologic therapy, age, and sex. Discrimination was quantified using the area under the receiver operating characteristic curve (AUC). Internal validation was performed using 500 bootstrap resamples with replacement to estimate optimism-corrected discrimination.

A two-sided *p* value < 0.05 was considered statistically significant. Statistical analyses were performed using SPSS version 25.0 (IBM Corp., Armonk, NY, USA) and R version 4.2.1 (R Foundation for Statistical Computing, Vienna, Austria).

## 3. Results

### 3.1. Study Population

A total of 84 patients were assessed for eligibility. Twelve were excluded due to recent antibiotic use, nine due to active infection, and four due to pregnancy. No patients were excluded due to known malignancy, severe organ failure, or recent initiation or modification of biologic therapy.

Among the remaining patients, 15 with Crohn’s disease were excluded from the present analysis to ensure a homogeneous cohort and the use of a validated histologic endpoint.

**Figure 1:**
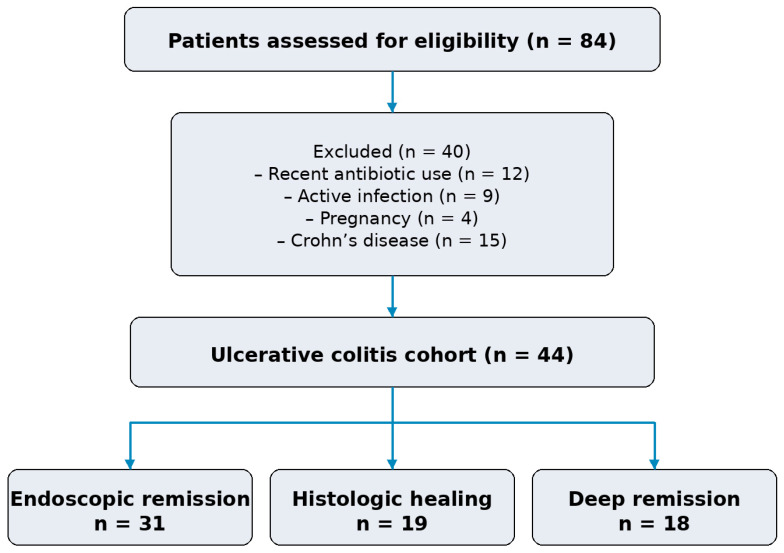
Flow diagram illustrating patient selection and final study cohort.

The final study population consisted of 44 consecutive patients with ulcerative colitis and complete endoscopic, histologic, and biomarker data (Figure 1).

Among included patients, 31 (70.5%) achieved endoscopic remission and 19 (43.2%) demonstrated histologic healing. In the ulcerative colitis cohort, deep remission was observed in 18/44 (40.9%). Baseline characteristics are presented in Table 1.

### 3.2. Baseline Characteristics According to Deep Remission Status

Baseline characteristics stratified by deep remission status were analyzed in the ulcerative colitis cohort and are presented in Table 2.

In the ulcerative colitis cohort, patients achieving deep remission had significantly lower CRP levels (*p* = 0.005) and significantly higher IL-10 concentrations (*p* < 0.001) compared with those without deep remission. Age, sex distribution, smoking status, and disease duration did not differ significantly between groups. Treatment exposure differed across groups (*p* = 0.039), with a higher proportion of patients receiving 5-ASA among those in deep remission.

### 3.3. Endoscopic–Histologic Distribution

The relationship between endoscopic and histologic status was evaluated in the ulcerative colitis cohort.

In the ulcerative colitis cohort (Table 3) histologic–endoscopic discordance was observed in 14 patients (31.8%), including 13 patients in endoscopic remission with persistent histologic activity and 1 patient with endoscopic activity but histologic healing. The association between endoscopic and histologic status was statistically significant (Pearson χ^2^ test, *p* < 0.001).

### 3.4. Distribution of Clinical Outcomes

The distribution of clinical outcomes in the ulcerative colitis cohort is presented in Table 4. Endoscopic remission was observed in 31/44 patients (70.5%), histologic healing in 19/44 (43.2%), and deep remission in 18/44 patients (40.9%).

Serum IL-10 concentrations according to deep remission status are shown in Table 5. In ulcerative colitis, median IL-10 levels were significantly higher in patients with deep remission compared to those without (4.70 vs. 2.80 pg/mL, *p* < 0.001).

These descriptive findings provide context for the regression analyses presented in the following sections and should be interpreted in conjunction with the primary analysis.

### 3.5. Associations Between IL-10 and Deep Remission

In Firth penalized logistic regression, higher IL-10 levels were significantly associated with deep remission in both univariable and multivariable models (Table 6). This association remained statistically significant after adjustment for C-reactive protein and treatment exposure.

The estimated effect of IL-10 remained consistent after adjustment for treatment exposure, with odds ratios of similar magnitude across models (univariable OR 45.47 [95% CI 4.35–475.05] vs. primary multivariable OR 87.79 [95% CI 4.07–1895.56] vs. extended multivariable OR 44.88 [95% CI 3.78–532.60]), suggesting no evidence of substantial confounding by treatment exposure.

The magnitude of the estimated odds ratios was large, with wide confidence intervals, reflecting limited sample size and potential quasi-separation rather than a precisely estimated effect size. These findings should therefore be interpreted as indicating a strong directional association between IL-10 and deep remission rather than its exact magnitude.

In sensitivity analyses comparing multivariable models with and without adjustment for treatment exposure, log-transformed IL-10 remained significantly associated with deep remission, with effect estimates of broadly similar magnitude across models, although estimates were imprecise and should be interpreted cautiously (Table 7).

### 3.6. Distribution of IL-10 According to Deep Remission

**Figure 2:**
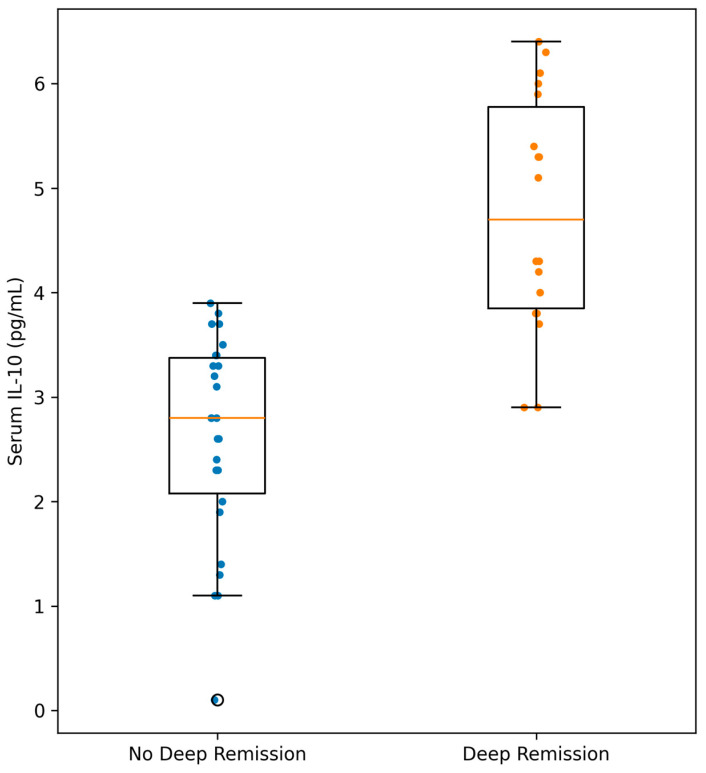
Serum interleukin-10 (IL-10) concentrations according to deep remission status. Blue dots represent individual patient values in the non–deep remission group, and orange dots represent individual patient values in the deep remission group. Values are presented as median and interquartile range. Differences between groups were assessed using the Mann–Whitney U test (*p* < 0.001).

Serum IL-10 concentrations according to deep remission status are illustrated in Figure 2.

### 3.7. Discriminatory Performance and Internal Validation

Model discrimination for deep remission was assessed and is presented in Table 8.

The univariable IL-10 model demonstrated apparent good discrimination (apparent AUC 0.84), which appeared to improve modestly in the primary multivariable model including CRP and biologic therapy (apparent AUC 0.88). The extended multivariable model including age and sex did not further improve discrimination (apparent AUC 0.87).

**Figure 3:**
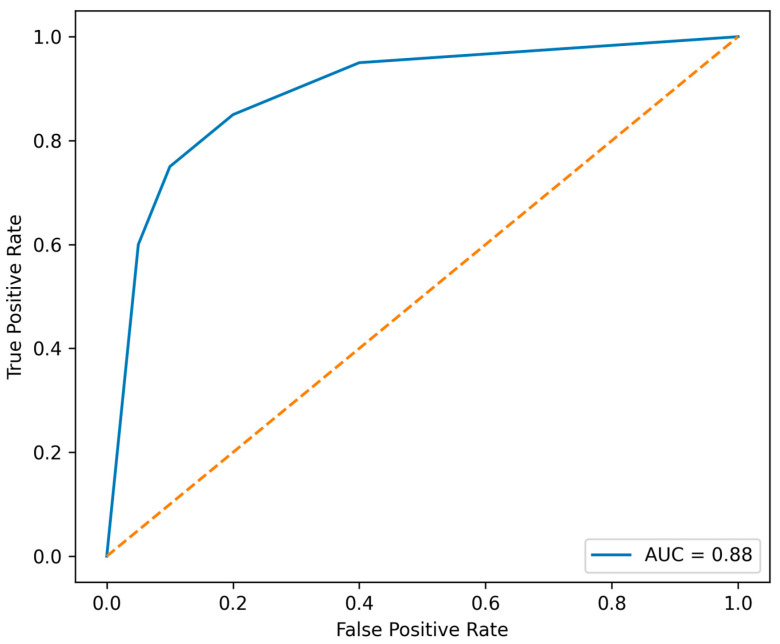
Receiver operating characteristic (ROC) curve for the primary multivariable model. The model included log-transformed interleukin-10 (IL-10), C-reactive protein (CRP), and biologic therapy exposure (biologic vs. non-biologic). The model demonstrated apparent good discrimination for deep remission, with an apparent area under the curve (AUC) of 0.88. The diagonal dashed line indicates chance discrimination.

Internal validation using bootstrap resampling (500 iterations) yielded optimism-corrected AUC estimates of 0.82, 0.85, and 0.84 for the univariable, primary, and extended models, respectively, suggesting relatively stable internal model performance; however, these estimates reflect internal validation and may be optimistic. The receiver operating characteristic (ROC) curve of the primary multivariable model is shown in Figure 3.

These findings suggest that IL-10 may be a key contributor to model discrimination within this cohort, with only modest incremental contribution from additional clinical variables. Discrimination estimates reflect internal model performance and should be interpreted cautiously given the sample size and the potential risk of overfitting.

### 3.8. Histologic Activity Within Endoscopic Remission

Among 31 patients in endoscopic remission, 13 (41.9%) had persistent histologic activity. The association between IL-10 detectability and histologic status was statistically significant (Fisher’s exact test, *p* < 0.001). In an exploratory analysis, the diagnostic performance of IL-10 detectability for identifying histologic activity among patients in endoscopic remission is summarized in Table 9.

IL-10 detectability demonstrated high sensitivity (92.9%) and negative predictive value (94.4%), with moderate specificity (77.3%) and positive predictive value (72.2%).

These findings suggest that IL-10 detectability may have potential utility in identifying patients with residual histologic activity despite endoscopic remission; however, given the limited sample size and the exploratory nature of this analysis, these results should be interpreted cautiously and considered hypothesis-generating.

## 4. Discussion

In this prospective observational study, serum IL-10 was independently associated with deep remission. Log-transformed IL-10 demonstrated a statistically significant association with the combined endpoint of endoscopic remission and histologic healing and retained statistical significance after adjustment for CRP, biologic therapy, and prespecified clinical covariates.

The primary multivariable model incorporating IL-10, CRP, and biologic therapy demonstrated apparent good discrimination with internal validation, suggesting that IL-10 may provide additional information within this cohort beyond conventional inflammatory markers.

Although IL-10 appeared to improve model discrimination as reflected by increases in AUC, such differences do not necessarily translate into improved clinical decision-making and should be viewed as supportive rather than definitive evidence of incremental value. The model also appeared to retain discrimination following bootstrap internal validation; however, these estimates reflect internal performance and may be optimistic. Calibration metrics were not formally assessed and should be considered when interpreting overall model performance [19,20,21,22].

Deep remission has emerged as a stringent therapeutic target, reflecting both macroscopic and microscopic resolution of inflammation [23,24,25]. However, reliable circulating biomarkers that capture mucosal immune quiescence remain limited. IL-10 is a central anti-inflammatory cytokine with well-established regulatory functions in intestinal immune homeostasis. Genetic defects in IL-10 signaling are causally linked to severe early-onset colitis, underscoring its biological relevance.

However, the biological interpretation of circulating IL-10 levels is not entirely straightforward. IL-10 is produced in response to inflammatory stimuli and may be elevated during active disease as part of a compensatory anti-inflammatory response. Therefore, increased systemic IL-10 concentrations do not necessarily indicate the absence of inflammation, but rather reflect the balance between pro-inflammatory and counter-regulatory immune pathways.

In addition, circulating IL-10 levels may not fully capture mucosal immune dynamics, as local cytokine production within the intestinal microenvironment may differ from systemic measurements. Previous studies have reported heterogeneous IL-10 profiles in active versus quiescent IBD, likely reflecting differences in disease phenotype, immune activation, and therapeutic exposure. As such, the relationship between systemic IL-10 and mucosal healing remains context-dependent and incompletely understood [26,27,28,29,30,31].

In the present study, higher IL-10 levels were observed in association with histologic healing, suggesting that detectable systemic IL-10 may reflect a dominant regulatory immune state in this specific clinical context. This observation should be considered hypothesis-generating and warrants further validation in studies integrating both systemic and mucosal immunologic assessments.

The magnitude of association observed in regression models was substantial, with wide confidence intervals reflecting limited sample size and event distribution rather than necessarily model instability. Firth penalized logistic regression was prespecified to mitigate small-sample bias and reduce the risk of quasi-separation. Accordingly, the results support the presence of an association, while acknowledging that the precise magnitude of effect remains imprecisely estimated. Given the relatively small sample size and the limited number of outcome events, the magnitude of the estimated odds ratios and the width of the corresponding confidence intervals warrant cautious interpretation and require confirmation in larger, adequately powered cohorts. In addition, the limited number of events relative to the number of predictors introduces a potential risk of model overfitting. Although Firth penalized regression and bootstrap internal validation were applied to mitigate this risk, some degree of optimism in model performance and inflation of effect estimates cannot be fully excluded.

A substantial proportion of IL-10 values were below the lower limit of detection. Modeling IL-10 as a continuous variable appeared to provide superior discrimination, suggesting potential additional informational value beyond simple detectability. Exploratory analysis using IL-10 detectability suggested a threshold effect, with detectable IL-10 showing an association with deep remission. These analyses were intended to complement, but not replace, the primary continuous-variable modeling approach. These findings should be interpreted cautiously and suggest that the observed relationship is unlikely to be solely attributable to a binary threshold phenomenon, although this requires confirmation.

Among patients in endoscopic remission, non-detectable IL-10 identified persistent histologic activity with high sensitivity and negative predictive value; however, these findings arise from a small exploratory subgroup and should be interpreted with caution. These analyses were performed in a limited subset and are not intended to support standalone diagnostic use. Histologic activity despite endoscopic remission represents a clinically relevant phenomenon associated with increased relapse risk. The observed association between IL-10 detectability and histologic discordance may suggest potential relevance as a non-invasive adjunct in future biomarker-based monitoring strategies; however, prospective validation is required before any clinical use can be considered. Confidence intervals for these estimates were relatively wide, reflecting the limited sample size and number of events.

From a clinical perspective, IL-10 may be best viewed as a complementary biomarker rather than a replacement for established inflammatory markers such as C-reactive protein or fecal calprotectin. While CRP reflects systemic inflammation and fecal calprotectin provides a sensitive measure of intestinal neutrophilic activity, IL-10 may capture counter-regulatory immune responses associated with mucosal healing. The combined use of IL-10 with conventional biomarkers may therefore have the potential to offer a more comprehensive assessment of inflammatory and regulatory pathways in ulcerative colitis.

In the present study, the lower limit of detection (3.9 pg/mL) provided a pragmatic threshold for defining IL-10 detectability in exploratory analyses. Detectable IL-10 levels were associated with histologic healing, whereas non-detectable levels were strongly associated with persistent histologic activity. However, given the limited sample size and the absence of externally validated cut-off values, IL-10 is more appropriately modeled as a continuous biomarker, and the clinical relevance of specific thresholds requires validation in larger cohorts.

Therapeutic exposure represents a potential source of confounding in biomarker studies. In the present cohort, biologic therapy exposure was explored descriptively and in sensitivity analyses, and did not materially alter the association between IL-10 and deep remission. Effect estimates for IL-10 remained stable across models with and without treatment adjustment. Nevertheless, residual confounding cannot be fully excluded, particularly in observational settings. Larger multicenter studies with stratified analyses across therapeutic classes will be necessary to disentangle treatment effects from intrinsic immune regulation [32,33,34].

Several limitations should be acknowledged when interpreting these findings. The study was conducted at a single tertiary referral center and included a relatively modest number of events, which may limit generalizability and contribute to statistical imprecision despite the use of prespecified Firth penalized regression and bootstrap internal validation. Although discrimination appeared to remain stable after optimism correction, the absence of formal calibration metrics should be noted, and the magnitude of the reported odds ratios should be interpreted cautiously, as effect size inflation cannot be entirely excluded in smaller datasets. Given that the present analysis was restricted to a homogeneous ulcerative colitis cohort, formal assessment of effect modification by disease subtype (ulcerative colitis vs. Crohn’s disease) was not applicable. The previously noted concern regarding disease subtype interaction was addressed by design through exclusion of Crohn’s disease and avoidance of pooled analyses across biologically distinct entities.

The cross-sectional design precludes assessment of temporal relationships and does not allow evaluation of longitudinal predictive performance, including relapse risk or treatment response. External validation in independent and more diverse cohorts was not performed, and therefore the transportability of the proposed model across different clinical settings, disease phenotypes, and therapeutic exposures remains to be established. Histologic healing was defined using the Geboes score and reflects established definitions in ulcerative colitis; however, its performance in broader clinical settings requires further validation. Finally, as in all observational studies, residual confounding cannot be fully excluded despite adjustment for key clinical variables and sensitivity analyses. Nevertheless, the combination of consecutive enrollment, blinded histologic assessment, standardized biomarker measurement, prespecified modeling strategies, and internal validation supports the internal validity of the observed associations [35,36,37]. Future studies should be conducted in multicenter settings with larger sample sizes to enhance generalizability and statistical robustness. In addition, inclusion of both ulcerative colitis and Crohn’s disease populations, analyzed within appropriate disease-specific methodological frameworks, will be important to further elucidate the role of IL-10 across the spectrum of inflammatory bowel disease.

In summary, serum IL-10 concentrations were independently associated with deep remission in this well-characterized cohort and showed potentially promising internally validated discriminatory performance within this cohort. Within the constraints of a cross-sectional design, the findings are biologically plausible and suggest that IL-10 may have potential relevance within biomarker-based stratification frameworks. These results should be considered hypothesis-generating and interpreted with caution given the modest number of events and resulting statistical uncertainty.

## Figures and Tables

**Table 1:** Baseline characteristics of the ulcerative colitis cohort (n = 44).

Variable	UC (n = 44)
Demographic and clinical characteristics	
Age, years	48.0 (36.0–60.0)
Male sex, n (%)	26 (59.1%)
Disease duration, years	5.0 (2.0–10.0)
CRP, mg/L	0.55 (0.10–6.75)
IL-10, pg/mL	3.4 (2.6–4.2)
Smoking (current), n (%)	8 (18.2%)
Prior surgery, n (%)	0 (0.0%)
Clinical outcomes	
Histologic healing, n (%)	19 (43.2%)
Endoscopic remission, n (%)	31 (70.5%)
Deep remission, n (%)	18 (40.9%)
Ulcerative colitis (Montreal extent)	
E1 (proctitis), n (%)	4 (9.1%)
E2 (left-sided), n (%)	3 (6.8%)
E3 (extensive), n (%)	37 (84.1%)
Treatment	
No treatment, n (%)	5 (11.4%)
5-ASA, n (%)	23 (52.3%)
Biologic therapy, n (%)	12 (27.3%)
Combination therapy, n (%)	4 (9.1%)

Footnote: Values are presented as median (interquartile range) or number (%). Percentages are calculated within the ulcerative colitis cohort.

**Table 2:** Baseline Characteristics by Deep Remission Status in Ulcerative Colitis (n = 44).

Characteristic	Non–Deep Remission (n = 26)	Deep Remission (n = 18)	*p*-Value
Age, years	51.0 (32.0–63.0)	49.0 (39.8–55.8)	0.701
Disease duration, years	6.0 (2.0–10.0)	5.0 (2.3–6.8)	0.340
C-reactive protein, mg/L	2.5 (0.1–10.0)	0.1 (0.1–1.2)	0.005
Interleukin-10, pg/mL	2.8 (2.08–3.38)	4.7 (3.85–5.78)	<0.001
Male sex, n (%)	14 (53.8%)	12 (66.7%)	0.531
Current smoking, n (%)	4 (15.4%)	4 (22.2%)	0.690
Montreal classification, n (%)			0.343
E1	1 (3.8%)	3 (16.7%)	
E2	2 (7.7%)	1 (5.6%)	
E3	23 (88.5%)	14 (77.8%)	
Treatment exposure, n (%)			0.039
No treatment	5 (19.2%)	0 (0%)	
5-ASA	10 (38.5%)	13 (72.2%)	
Biologic therapy	8 (30.8%)	4 (22.2%)	
Combination therapy	3 (11.5%)	1 (5.6%)	

Footnote: Values are presented as median (interquartile range) or number (%). Continuous variables were compared using the Mann–Whitney U test and categorical variables using Fisher’s exact test or χ^2^ test, as appropriate. Percentages are calculated within the ulcerative colitis cohort.

**Table 3:** Association Between Endoscopic and Histologic Status in Ulcerative Colitis (n = 44).

	Histologic Activity	Histologic Healing	Total
Endoscopic Activity	12 (27.3%)	1 (2.3%)	13 (29.5%)
Endoscopic Remission	13 (29.5%)	18 (40.9%)	31 (70.5%)
Total	25 (56.8%)	19 (43.2%)	44 (100%)

Footnote: Values are presented as number (%). Percentages are calculated within the ulcerative colitis cohort. Pearson χ^2^ test (two-sided), *p* < 0.001.

**Table 4:** Distribution of endoscopic remission, histologic healing, and deep remission in the ulcerative colitis cohort.

Outcome	UC (n = 44)
Endoscopic remission, n (%)	31 (70.5%)
Histologic healing, n (%)	19 (43.2%)
Deep remission, n (%)	18 (40.9%)

Footnote: Values are presented as number (%). Percentages are calculated within the ulcerative colitis cohort. Histologic healing was defined using the Geboes score.

**Table 5:** Serum IL-10 concentrations according to deep remission status in ulcerative colitis.

Status	IL-10, Median (IQR), pg/mL	*p*-Value
No deep remission	2.80 (2.08–3.38)	
Deep remission	4.70 (3.85–5.78)	<0.001

Footnote: Values are presented as median (interquartile range).

**Table 6:** Firth-penalized logistic regression analysis for deep remission in ulcerative colitis (Primary Analysis with Adjustment for Treatment Exposure).

Analysis	Variable	Odds Ratio (95% CI)	*p*-Value
Univariable model	log(IL-10)	45.47 (4.35–475.05)	0.001
Primary multivariable model	log(IL-10)	87.79 (4.07–1895.56)	0.004
	CRP	0.82 (0.64–1.06)	0.132
	Biologic therapy	0.29 (0.03–2.50)	0.260
	E2 vs. E1	2.39 (0.009–652.5)	0.761
	E3 vs. E1	3.26 (0.07–158.1)	0.550
Extended multivariable model	log(IL-10)	44.88 (3.78–532.60)	0.003
	CRP	0.92 (0.73–1.16)	0.451
	Biologic therapy (vs non-biologic)	0.24 (0.03–1.89)	0.177
	Age	1.01 (0.96–1.07)	0.693
	Male sex	1.68 (0.28–10.02)	0.565

Footnote: Odds ratios (ORs) with 95% confidence intervals (CIs) were estimated using Firth penalized logistic regression. The primary multivariable model included log-transformed interleukin-10 (IL-10), C-reactive protein (CRP), and biologic therapy exposure (biologic vs. non-biologic), which was included a priori to address potential confounding. Disease extent (E1/E2/E3) was included to account for within-disease heterogeneity. The extended model additionally included age and sex.

**Table 7:** Sensitivity analysis of the association between IL-10 and deep remission with and without treatment adjustment in ulcerative colitis.

Model	Variable	OR (95% CI)	*p*-Value
Multivariable model without treatment adjustment	log(IL-10)	142.60 (5.10–3985.00)	0.003
	CRP	0.88 (0.69–1.12)	0.298
	E2 vs. E1	0.72 (0.02–28.50)	0.862
	E3 vs. E1	1.41 (0.05–38.20)	0.812
Multivariable model with treatment adjustment (primary model, Table 6)	log(IL-10)	87.79 (4.07–1895.56)	0.004
	CRP	0.82 (0.64–1.06)	0.132
	Biologic therapy	0.29 (0.03–2.50)	0.260
	E2 vs. E1	2.39 (0.009–652.5)	0.761
	E3 vs. E1	3.26 (0.07–158.1)	0.550

Footnote: Odds ratios (ORs) with 95% confidence intervals (CIs) were estimated using Firth penalized logistic regression. The multivariable model without treatment adjustment included log-transformed interleukin-10 (IL-10), C-reactive protein (CRP), and disease extent (Montreal classification: E1/E2/E3), thereby mirroring the structure of the primary model except for treatment exposure. The multivariable model with treatment adjustment corresponds to the prespecified primary multivariable model presented in Table 6. Across both models, log-transformed IL-10 remained significantly associated with deep remission, with effect estimates of similar magnitude. However, given the limited number of events relative to the number of covariates, there is a potential risk of overfitting, and effect estimates were imprecisely estimated, as reflected by the wide confidence intervals. These findings should therefore be interpreted cautiously and considered exploratory.

**Table 8:** Discrimination Performance of Models for Deep Remission.

Model	Apparent AUC	Optimism-Corrected AUC
Univariable (IL-10)	0.84	0.82
Primary multivariable	0.88	0.85
Extended multivariable	0.87	0.84

Footnote: Apparent AUC represents model discrimination in the original dataset. Optimism-corrected AUC was estimated using 500 bootstrap resamples to account for potential overfitting. The univariable model included log-transformed interleukin-10 (IL-10) as the sole predictor. The primary multivariable model included log-transformed IL-10, C-reactive protein (CRP), and biologic therapy exposure (biologic vs. non-biologic). The extended multivariable model additionally included age and sex. These analyses reflect internal model performance within the UC cohort and should not be interpreted as evidence of external validity or generalizability.

**Table 9:** Diagnostic performance of IL-10 detectability for histologic activity among patients in endoscopic remission.

Metric	Value
Sensitivity	92.9% (95% CI, 68.5–98.7)
Specificity	77.3% (95% CI, 56.6–89.9)
PPV	72.2% (95% CI, 49.1–87.5)
NPV	94.4% (95% CI, 74.2–99.0)

Footnote: Values are presented as percentages with 95% confidence intervals (Wilson method), calculated from observed proportions. Non-detectable IL-10 levels (<3.9 pg/mL) were considered indicative of histologic activity.

## Data Availability

The datasets generated during the current study are available from the corresponding author on request. Data are not publicly available due to institutional and ethical restrictions.

## Supplementary Materials

The following supporting information can be downloaded at: https://www.mdpi.com/article/10.3390/biomedicines14040908/s1, Table S1: STROBE checklist.

## Abbreviations

The following abbreviations are used in this manuscript:

UC	Ulcerative colitis
IL-10	Interleukin-10
CRP	C-reactive protein
ROC	Receiver operating characteristic
AUC	Area under the curve
LOD	Limit of detection
OR	Odds ratio
CI	Confidence interval
EPV	Events per variable
IQR	Interquartile range
