# Supplementary material for: Serum Interleukin-10 as a Potential Biomarker for Deep Remission and Histologic Activity in Ulcerative Colitis
Source: Biomedicines. 2026 Apr 16;14(4):908. doi: 10.3390/biomedicines14040908 (PMC13113510; doi:10.3390/biomedicines14040908)
Supplement: Supplementary file 1 [file biomedicines-14-00908-s001.zip › biomedicines-4206652-supplementary.pdf]

Table S1. STROBE Statement—Checklist of items that should be included in reports of ***cross-sectional studies***

	Item No	Recommendation	Reported on Page/Section
**Title and abstract**	1	(*a*) Indicate the study's design with a commonly used term in the title or the abstract	Title; Abstract (p.1)
		(*b*) Provide in the abstract an informative and balanced summary of what was done and what was found	Abstract (p.1)
**Introduction**			
Background /rationale	2	Explain the scientific background and rationale for the investigation being reported	Introduction (pp. 2–3)
Objectives	3	State specific objectives, including any prespecified hypotheses	Introduction (final paragraph, p.3)
**Methods**			
Study design	4	Present key elements of study design early in the paper	Methods, Section 2.1 (p.4)
Setting	5	Describe the setting, locations, and relevant dates, including periods of recruitment, exposure, follow-up, and data collection	Methods, Sections 2.1–2.2 (pp.4–5)
Participants	6	(*a*) Give the eligibility criteria, and the sources and methods of selection of participants. Describe methods of follow-up	Methods, Section 2.1 (pp.4–5)
		(*b*) For matched studies, give matching criteria and number of exposed and unexposed	Not applicable (no matched design)
Variables	7	Clearly define all outcomes, exposures, predictors, potential confounders, and effect modifiers. Give diagnostic criteria, if applicable	Methods, Sections 2.4–2.10 (pp.5–8)
Data sources/ measurement	8*	For each variable of interest, give sources of data and details of methods of assessment (measurement). Describe comparability of assessment methods if there is more than one group	Methods, Sections 2.3–2.6 and 2.8–2.9 (pp.5–7)
Bias	9	Describe any efforts to address potential sources of bias	Methods (Sections 2.1, 2.4–2.6, 2.10: consecutive enrollment, blinding, multivariable adjustment); Discussion (Strengths and Limitations)
Study size	10	Explain how the study size was arrived at	No formal sample size calculation was performed, as this was an observational study based on consecutive patient enrollment within a predefined study period. The available sample size therefore reflects the total number of eligible patients during the study timeframe and is acknowledged to limit statistical power, particularly for subgroup and interaction analyses.
Quantitative variables	11	Explain how quantitative variables were handled in the analyses. If applicable, describe which groupings were chosen and why	Methods, Sections 2.5 and 2.10 (pp.5–8)
Statistical methods	12	(*a*) Describe all statistical methods, including those used to control for confounding	Methods, Section 2.10 (multivariable Firth penalized logistic regression, including CRP, biologic therapy, and disease extent as covariates)
		(*b*) Describe any methods used to examine subgroups and interactions	Methods, Section 2.10 (Exploratory subgroup analyses within endoscopic remission)

		(*c*) Explain how missing data were addressed	Methods, Section 2.1 (p.4; no missing data observed)
		(*d*) If applicable, explain how loss to follow-up was addressed	Not applicable (cross-sectional study; no follow-up)
		(*e*) Describe any sensitivity analyses	Results, Section 3.5 (pp.12–13)
**Results**			
Participants	13*	(a) Report numbers of individuals at each stage of study—eg numbers potentially eligible, examined for eligibility, confirmed eligible, included in the study, completing follow-up, and analysed	Results, Section 3.1 (pp.9–10)
		(b) Give reasons for non-participation at each stage	Not applicable (consecutive enrollment; no non-participation or attrition)
		(c) Consider use of a flow diagram	Figure 1
Descriptive data	14*	(a) Give characteristics of study participants (eg demographic, clinical, social) and information on exposures and potential confounders	Results, Tables 1–3 (pp.9–11)
		(b) Indicate number of participants with missing data for each variable of interest	Methods Section 2.1; Results (complete-case dataset)
		(c) Summarise follow-up time (eg, average and total amount)	Not applicable (cross-sectional design; no follow-up period)
Outcome data	15*	Report numbers of outcome events or summary measures over time	Results, Section 3.1; Table 1 (pp.9–10)
Main results	16	(*a*) Give unadjusted estimates and, if applicable, confounder-adjusted estimates and their precision (eg, 95% confidence interval). Make clear which confounders were adjusted for and why they were included	Methods Section 2.10 (model specification and covariate selection rationale); Results Tables 8–10
		(*b*) Report category boundaries when continuous variables were categorized	Methods, Section 2.5 (pp.5–6); Results, Section 3.6 (p.13)
		(*c*) If relevant, consider translating estimates of relative risk into absolute risk for a meaningful time period	Not applicable (cross-sectional logistic analysis; no time-to-event or absolute risk estimation)
Other analyses	17	Report other analyses done—eg analyses of subgroups and interactions, and sensitivity analyses	Results, Sections 3.4–3.6 (pp.11–14)
**Discussion**			
Key results	18	Summarise key results with reference to study objectives	Discussion (first paragraph, p.16)
Limitations	19	Discuss limitations of the study, taking into account sources of potential bias or imprecision. Discuss both direction and magnitude of any potential bias	Discussion (Strengths and Limitations section, pp.18–19)
Interpretation	20	Give a cautious overall interpretation of results considering objectives, limitations, multiplicity of analyses, results from similar studies, and other relevant evidence	Discussion (pp.16–19)
Generalisability	21	Discuss the generalisability (external validity) of the study results	Discussion (Strengths and Limitations section, pp.18–19)
**Other information**			
Funding	22	Give the source of funding and the role of the funders for the present study and, if applicable, for the original study on which the present article is based	Funding section (p.20)

*Give information separately for exposed and unexposed groups.

Note: An Explanation and Elaboration article discusses each checklist item and gives methodological background and published examples of transparent reporting. The STROBE checklist is best used in conjunction with this article (freely

available on the Web sites of PLoS Medicine at http://www.plosmedicine.org/, Annals of Internal Medicine at http://www.annals.org/, and Epidemiology at http://www.epidem.com/). Information on the STROBE Initiative is available at http://www.strobe-statement.org.
